# Dysgerminoma in three patients with Swyer syndrome

**DOI:** 10.1186/1477-7819-5-71

**Published:** 2007-06-23

**Authors:** Nadereh Behtash, Mojgan Karimi Zarchi

**Affiliations:** 1 Gynecology Oncology Department, Vali-Asr Hospital, Keshavarz Blvd., Tehran 14194, Iran

## Abstract

**Background:**

Dysgerminoma is the most common malignant germ cell tumor of the ovary. This malignancy can be associated with pure gonadal dysgenesis or Swyer syndrome, mixed gonadal dysgenesis and partial gonadal dysgenesis.

**Case presentation:**

Dysgerminoma developed in 3 phenotypic female patients with 46 XY pure gonadal dysgenesis. All patients presented first with abdominopelvic mass. Laparatomy was done. 46 XY karyotype was made by lymphocyte culture. Then these patients underwent gonadectomy that histopathology results were streak ovaries without evidence for malignancy. Two patients received postoperative adjuvant therapy.

**Conclusion:**

In Patients with Swyer syndrome the risk of dysgerminoma is high and gonadectomy is recommended. Also 5% of dysgerminomas are discovered in phenotypic female and 46 XY karyotype, thus in adolescent with dysgerminoimas and amenorrhea, karyotype should be done.

## Background

Since 1955, when Swyer [1,2] first described two phenotypic women with gonadal dysgenesis without the stigma of Turner syndrome (46, XY pure gonadal dysgenesis of Swyer syndrome), several authors have reported over **74** tumoral growths of their dysgenetic gonads [3-19].

The propensity of tumor development in Swyer syndrome is significant, a incidence of 20–30% is reported. The most common tumor is the often-bilateral gonadoblastoma, but dysgerminoma and even embryonal carcinoma also seen [2]. Approximately 5% of dysgerminomas are discovered in phenotypic females with abnormal gonads and 46 XY karyotype [1].

We present three patients with pelvic mass, of which two patients, in spite of primary amenorrhea had nearly complete secondary sex characteristic. Another patient had secondary amenorrhea. All the patients had dysgerminoma, that underwent unilateral salpingoopherectomy and two of these, received adjuvant therapy. Gonadectomy after diagnosis of XY karyotype was done.

## Case presentation

### Case 1

A 20 years old girl (160 cm height and 57 kg weight) presented in September 1999 with 6 months history of lower abdominal pain and gradual distention of abdomen. Clinically, she was noted to have an abdominal mass extending 4 cm above umbilicus. She had undergone an ultrasonography, which showed a huge complex abdominopelvic mass arising from the right ovary. Additional evaluation was negative for serum βHCG and AFP, but CA125 was 110 IU/ml. The FSH was 34 mIU/ml, the LH was 28 mIU/ml and serum estradiol was 36 pg/ml. Her past medical history was negative except for primary amenorrhea. She had gradual breast development since eleven years old and at her 1^st ^visit, she had nearly complete development of secondary sex characteristics. On examination the abdomen was soft, non tender with a palpable nontender fixed abdominopelvic mass. There was no evidence of ascitis. In vaginal exam, there was a normal length vagina and cervix. Uterus was palpable in rectovaginal exam.

**Figure 1:**
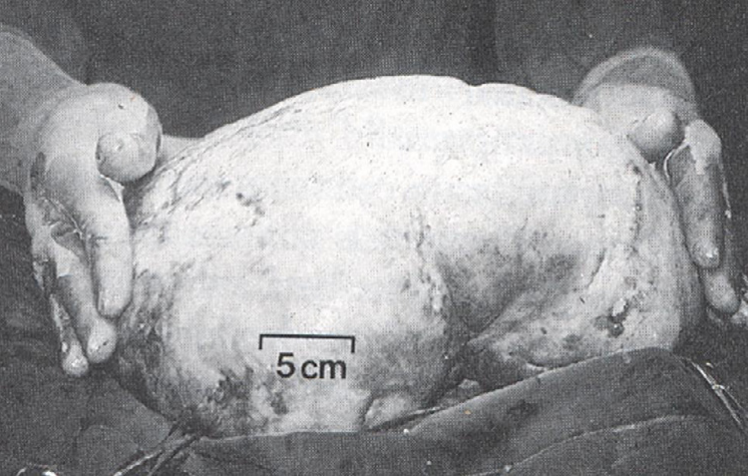
Dysgerminoma. An usually large tumor, showing a smooth boss elated external surface.

Exploratory laparotomy revealed a normal (infantile) uterus and tubes and a huge abdominopelvic mass 25 cm diameter and 3100 g weight, which was resected (figure 1). Gonads on the contralateral side were normal.

**Figure 2:**
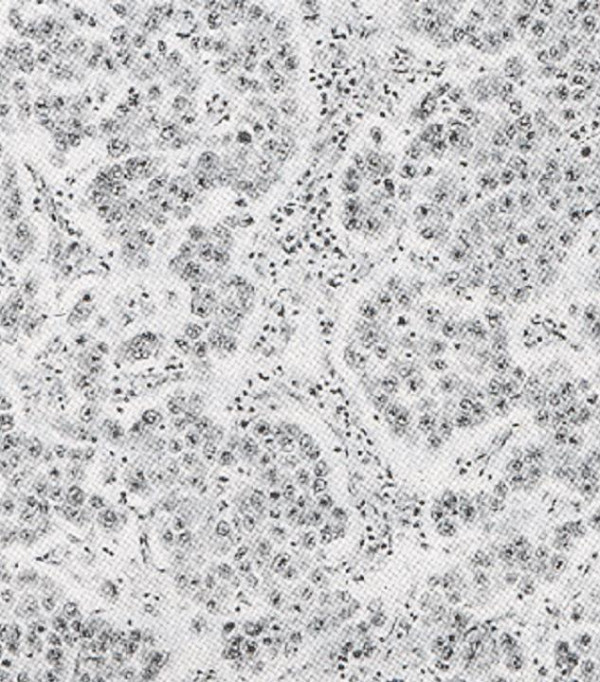
Dysgerminoma. The clamps of tumor cells are separated by fibrous stromal strands, which are infiltrated by inflammatory cells.

Mass resection was performed and uterus was preserved. The cut surface of the tumor was soft; light gray with focal areas of hemorrhage. Pathology revealed ovarian dysgerminoma of the right ovary characterized by neoplastic cells that had round to ovoid or slightly irregular nuclei with coarse clumped chromatin and prominent nucleoli. Mitoses were numerous. The cytoplasm was clear to lightly eosenophyilic and cell borders were well defined. Aggregation of tumor cells was separated by fibrous septa that contained scattered lymphocytes (figure 2). The tumor was classified as stage IIIC.

Full metastatic work-up was negative. Karyotype was consistent with 46 XY (nonmosaic). At the end of the second course of chemotherapy (Bleomycin plus etoposide plus cisplatin), liver enzyme rose significantly and jaundiced, and then due to probable drugs induced cholestatic hepatitis, she was referred to radiotherapy. She received 4000 cGy and become severely leucopenic. One-month later exploration laparoscopy was performed and one residual gonad or streak gonad was found at the opposite site and she underwent gonadectomy. Histopathological examination showed a streak ovary without evidence for malignancy. She received estrogen and progesterone postoperatively, until now and she has monthly menstrual flow.

### Case 2

**A **19 years old female G_0_P_0 _(159 cm high and 55 kg Wight) with primary amenorrhea was referred to the Tehran University Gynecology cancer center with 7 months history of abdominal pain, which progressed to abdominal distension. Evaluation included pelvic exam and ultrasound, which documented a solid complex mass of 123 × 80 mm size. The first sonographic diagnosis was hematometrocolpos or endometriosis. Computerized tomography scan confirmed no upper abdominal disease or lymphadenopathy and confirmed pelvic mass and no ascitis. Additional evaluation was normal for serum CA125 and AFP and βHCG. FSH was 80 mIU/ml, LH was 38 mIU/ml, estradiol was 29 pg/ml and LDH was 1450 IU/ml. Her past medical history was negative. Her karyotype was reported as 46 XY, thus she underwent laparotomy, peritoneal washing, resection of tumor (in right gonad), Para aortic and bilateral lymphadenectomy and staging biopsies were done. Infantile uterus preserved and left gonad was resected. The right ovary measured 120 mm and weight 1700 g. Pathology revealed dysgerminoma in right ovary and Tumor was classified as stage IIB. Histological examination of left ovary was infantile gonad including fibrous bands that don't seen malignancy.

Adjuvant chemotherapy consisted of 6 cycle of BEP after surgery was administered. Chest X-ray, abdominal and pelvic ultrasound and biochemical markers after 4 years remain normal. She receives estrogen and progesterone and has menstrual flow.

### Case 3

A 17 years old girl (168 cm high and 59 kg weight) with secondary amenorrhea was referred to our center in October 2004 with 4 months history of abdominal pain and distention of abdomen. Evaluation prior to referral included an ultrasound, which documented a complex abdominopelvic mass measured 95 × 80 mm thought to arise from the right ovary. Tumor markers were evaluated, that serum BHCG and AFP was normal, but CA125 was 90 IU/ml, LDH 844 IU/ml FSH 45 mIU/ml, LH 27 mIU/ml and estradiol 44 pg/ml. Past medical history in this patient was negative, except for secondary amenorrhea. She had breast development (tanner4) since 13 years old and at her 1^st ^visit she had nearly complete development of secondary sex characteristic. In rectal exam, there was a normal length vagina and cervix, and uterus was palpable. Laparatomy revealed a normal uterus and tubes and a large pelvic mass, which resected. In left side there was no gonadal lesion. Mass resection was performed. Uterus was preserved. Pathology revealed ovarian dysgerminoma of the right ovary. Full metastatic work-up was negative. Karyotype was consistent with 46 XY (pure). Due to tumor was classified as stage IA grade 1, adjuvant therapy was not commented. The patient admitted for unilateral gonadectomy. Histopathological examination of left ovary showed a streak ovary that displays ovarian stroma but no follicles. She had been followed for 18 months and with estrogen plus progesterone had menses out flow.

## Discussion

Gonadal dysgenesis is characterized by the histology of the gonads of affected patient and based on that the 46 XY gonadal dysgenesis may be divided in to 3 histological categories:

1) Complete or pure gonadal dysgenesis or Swyer syndrome. The patients are phenotypic females with a 46 XY karyotype and hypoplastic gonads without germ cells. They present most often with primary amenorrhea with normal stature. The gonads are usually streaks, but there may be some development of secondary sexual characteristics as well as a few episodes of uterine bleeding [1,2].

2) Mixed gonadal dysgenesis that primary amenorrhea is associated with various mosaic status, the most common of which is 45X/46xx. When compared to the pure 45X cell line, individuals with 45X/46XX are taller and have fewer abnormalities. Spontaneous menstruation occurs in approximately 20% of these patients.

3) Partial gonadal dysgenesis. They have karyotype 46XX with part of one of the X chromosomes missing. The phenotype is variable depending on the amount and location of the missing genetic material. In 46 XY partial gonadal dysgenesis individuals there is some testicular development; therefore they present as newborns with ambiguous genitalia.

The etiology of 46 XY gonadal dysgenesis is though to be a short arm Y chromosome deletion involving SRY, a mutation in other genes that leads to inhibition of SRY function or mutation of SRY function [2].

To date, 20% of 46 XY pure gonad dysgenesia are explained by a mutation or a deletion in SRY. In 80%, SRY is apparently normal. A female patient with an XY Karyotype who has a Palpable mullerian system, normal female testosterone levels, and lack of sexual development has Swyer syndrome, Tumor transformation in the gonadal ridge can occur at any age [1-3].

The incidence of neoplasia in patients with gonadal dysgenesis is wider than reported. In 50 reported cases, there were 11 malignancy, 15 adenoma and 10 benign cases; a 22% incidence of malignancy and a 52% incidence of neoplasia. More recent series indicate a lower overall incidence of gonadal tumors about 5–10% but in Swyer syndrome the risk of gonadal neoplasia is high (20–30%), dictating early prophylactic removal of these dysgenetic gonads (2). Also Slowikowska [7] reported, neoplasia may be occurred in 16.7% to 23.1% of patients with gonadal dysgenesis.

Uehara *et al*., and Amice showed that SRY may play a formation of gonadal tumors, especially dysgerminoma in Swyer syndrome [20,21]. However intra abdominal gonads should be removed as early in life as possible because of the known risk of tumor development [2].

The patients with 46 XY gonadal dysgenesis patients are diagnosed in early adolescence with delayed pubertal development. As expected they show elevated gonadotropins, normal female levels of androgens and low levels of estrogens, female external genitalia, uterus and fallopian tubes. Minimal breast enlargement reflects peripheral aromatization of androgens. Menstrual function suggests tumor development in the streak gonad. These streaks often display ovarian stroma but no follicles.

In our cases, gonadotropins were about postmenopausal level (FSH>20 IU/L) and estradiol was at the low end of normal (40–400 pg/ml) in reproductive ages. Also menstrual function and breast enlargement associated with gonadotropin – independent precocious puberty. In this patient, estrogen or androgens produced by ovarian tumor [22].

In accordance with Scully view the germ cells of the dysgerminoma seem the most likely source of the HCG, whereas the increased androgens were probably produced by the cells of the gonadoblastoma. Both hormone levels fell markedly on complete removal of the gonadal masses [19]. But in our cases BHCG levels were normal and testosterone was not measured before surgery.

Estrogen and progestin sequential therapy supports female secondary sex development in patients with gonadal dysgenesis and they are potential candidate for donor oocyte [1,2].

Kawai *et al*., analyzed seven tumor markers in germ cell tumors of the ovary. He showed positive rate of CA125 was over 50% in all tumor types except mature cystic teratoma. So CA125 was useful for the screening of malignant germ cell tumors. Dysgerminoma had a high positive rate of LDH. Positive rate of AFP was 11.8% in dysgerminoma, 100% in yolk sac tumor and 61.9% in immature teratoma [23].

In this article the plasma levels of CA125 in cases 1 and 3 were higher than expectant range, but in case2 were normal. LDH in case 2 and 3 were high and AFP in all patients was normal.

Dysgerminoma is the most common malignant germ cell tumor of the ovary. It can be found either in a pure form or mixed with other germinal elements. Therefore in premenarchal patients with a pelvic mass, the karyotype should be determined. About 65% of dysgerminomas are stage I at diagnosis. About 85–90% of stage I tumors are confined to one ovary; 10–15% is bilateral. Dysgerminoma is the only germ cell malignancy that has this significant rate of bilaterality, other germ cell tumors being rarely bilateral [1].

 The treatment of patient with early disgerminoma is primarily surgical, including resection of the primary lesion and proper surgical staging. Chemotherapy and/or radiation are administered to patients with metastatic disease. In patients whose contra lateral ovary has been preserved, disease can develop in 5% to 10% of the retained gonads over the next 2 years [1].

These three cases presented demonstrate the occurrence of dysgerminoma in patients with Swyer syndrome. There is a relative paucity of published case studies on dysgerminoma in female patients with pure dysgenetic gonad. The development of secondary sex characteristics in these three patients is most unusual. The sexual development were complete and only along females lines, furthermore these patients had withdrawal bleeding response to estrogen and progesterone. A small number of cases have reported that unusual female secondary sexual development in patients with 46 XY gonadal digenesis [24].

## Conclusion

Bilateral dysgenesis of the testes (Swyer syndrome) affected individuals has an XY karyotype but normal (infantile) female external and internal genitalia. In these patients the risk of dysgerminoma is high and gonadectomy was recommended. Also in 5% of cases of dysgerminoma, XY karyotype is seen. Thus in adolescents patients with dysgerminoma with primary amenorrhea and secondary amenorrhea, Karyotype should be done. Also menstrual function in patients with 46 XY karyotype may be associated with estrogen secretion of tumoral lesion and investigation of gonads is recommended.

## Competing interests

The author(s) declare that they have no competing interests.

## Authors' contributions

NB: conception and editing of manuscript

MKZ: Literature search, and preparation of manuscript

All authors read and approved final manuscript.
